# Frequency of Neurological Presentations of Coronavirus Disease in Patients Presenting to a Tertiary Care Hospital During the 2019 Coronavirus Disease Pandemic

**DOI:** 10.7759/cureus.9846

**Published:** 2020-08-18

**Authors:** Samar Iltaf, Meraj Fatima, Salma Salman, Jawwad-us Salam, Saira Abbas

**Affiliations:** 1 Neurology, Dow International Medical College, Dow University of Health Sciences, Karachi, PAK; 2 Medicine, Dow International Medical College, Dow University of Health Sciences, Karachi, PAK; 3 Neurology, Dow International Medical College, Dow University Hospital, Dow University of Health Sciences, Karachi, PAK

**Keywords:** coronavirus disease 2019 (covid-19), polymerase chain reaction (pcr), cerebro-vascular accident (stroke), guillain-barre syndrome (gbs)

## Abstract

Background

Coronavirus disease 2019 (COVID-19), caused by infection with the severe acute respiratory syndrome coronavirus 2 (SARS-CoV-2), usually presents clinically with cough, fever, shortness of breath, and loss of taste and/or smell. COVID-19 can also present with neurologic signs and symptoms, including headache, hyposmia/anosmia, encephalopathy, meningoencephalitis, Guillain-Barré syndrome, stroke, and seizure. Viral transmission occurs through aerosols generated when an infected person coughs, sneezes, or exhales and by direct touching of contaminated surfaces. The present study evaluated the frequency of neurologic presentations of coronavirus disease in patients presenting at a tertiary care hospital during the COVID-19 pandemic.

Methodology

This cross-sectional study included 350 inpatients and outpatients (self-isolated) with polymerase chain reaction-confirmed SARS-CoV-2 infection who presented at Dow International Medical College of Karachi between March and June 2020. Of these 350 patients, 68 (18.9%) presented with neurological signs and symptoms and were further evaluated. The data were analyzed statistically using IBM Statistical Product and Service Solutions (SPSS) for Windows, version 20.0 (IBM Corp., Armonk, NY).

Results

The 350 patients with SARS-CoV-2 infection included 245 (70%) men and 105 (30%) women; of these, 262 (74.9%) were married, and 88 (25.1%) were unmarried. Patients ranged in age from 17 to 88 years (mean ± standard deviation, 49.5 ± 17.4 years), with 68 (18.9%) having neurological manifestations. Headache was the most frequent neurological symptom, reported in 21 (6%) patients, followed by vertigo in 12 patients (3.4%), numbness/paresthesia in 11 (3.1%), altered level of consciousness in seven (2%), hyposmia/anosmia in five (1.4%), and encephalitis in three (0.9%). Other symptoms included sudden hemiparesis (stroke) in two patients (0.6%), flaccid paralysis due to Guillain-Barre syndrome in one (0.3%), and seizure in one (0.3%).

Conclusion

Neurological involvement is not infrequent in patients with COVID-19. Neurologic manifestations should be carefully monitored in infected patients. COVID-19 should be suspected in patients presenting with neurological abnormalities and should be included in the differential diagnosis to prevent further virus transmission.

## Introduction

The severe acute respiratory syndrome coronavirus 2 (SARS-CoV-2) is a ribonucleic acid (RNA) virus belonging to the family coronaviridae that is transmitted via respiratory aerosols, fomites, and directly from person to person. Coronavirus disease 2019 (COVID-19) was declared a worldwide pandemic by the World Health Organization on March 11, 2020 [1]. COVID-19 was first diagnosed in the city of Wuhan, China [2]. The pulmonary system is the most commonly affected, with symptoms including shortness of breath, fever, and cough the most commonly reported features. Observational studies have suggested that COVID-19 may have neurologic manifestations, including headache, nausea, vomiting, myalgia, dizziness, hyposmia/anosmia, encephalitis, and impaired consciousness (encephalopathy) [3,4]. Although the exact mechanism by which SARS-CoV-2 enters the central nervous system has not been determined, it may spread directly from the cribriform plate through the blood circulation, via mechanisms that include free radical or immune-mediated injury [4].

This study aimed to evaluate the frequency of neurologic presentations of coronavirus disease in patients presenting at a tertiary care hospital during the COVID-19 pandemic.

## Materials and methods

This cross-sectional study assessed the frequency of various neurological presentations of COVID-19 in patients treated for this disease at Dow University of Health Sciences (DUHS), a public sector tertiary care teaching hospital at Karachi, Pakistan, from March to June 2020. All patients, both inpatients and outpatients, who had oropharyngeal or nasopharyngeal swabs polymerase chain reaction (PCR)-positive for SARS-CoV-2, were recruited. Patients positive for immunoglobulin G antibodies to SARS-CoV-2 were excluded, as were patients with prior neurological or psychiatric diseases, systemic malignancy, hypercoagulability state, intracranial tumors, uncontrolled hypertension or diabetes mellitus, extremes of ages or anemia. The study protocol was approved by the ethics committee of the university, and all patients provided written informed consent.

A survey on neurological manifestations was specially designed for COVID-19 patients by researchers in the clinical faculty of the neurology department. This subjective survey addressed 10 neurological manifestations of COVID-19: headache, altered sensation, nausea and vomiting, sudden hemiparesis (stroke), numbness and paresthesia, vertigo, ataxia, seizure, encephalitis/meningitis, Guillain-Barré Syndrome (GBS), and myelitis. Neurological manifestations were later confirmed by a thorough review of all available patient records.

The sample size was calculated using the RaoSoft® Sample Size Calculator (RaoSoft, Inc., Seattle, WA), based on a 36.4% response distribution, a confidence interval of 95%, and a margin of error of 5%, resulting in a sample size of 350 patients. Statistical analyses were performed using IBM Statistical Product and Service Solutions (SPSS) Statistics for Windows, version 20.0. (IBM Corp., Armonk, NY). Quantitative variables were summarized as mean and standard deviation (SD) and qualitative variables as frequency and percentage. Qualitative variables were cross-tabulated with age group and gender and compared by Chi-square tests. A p-value <0.05 was considered statistically significant.

## Results

**Figure 1:**
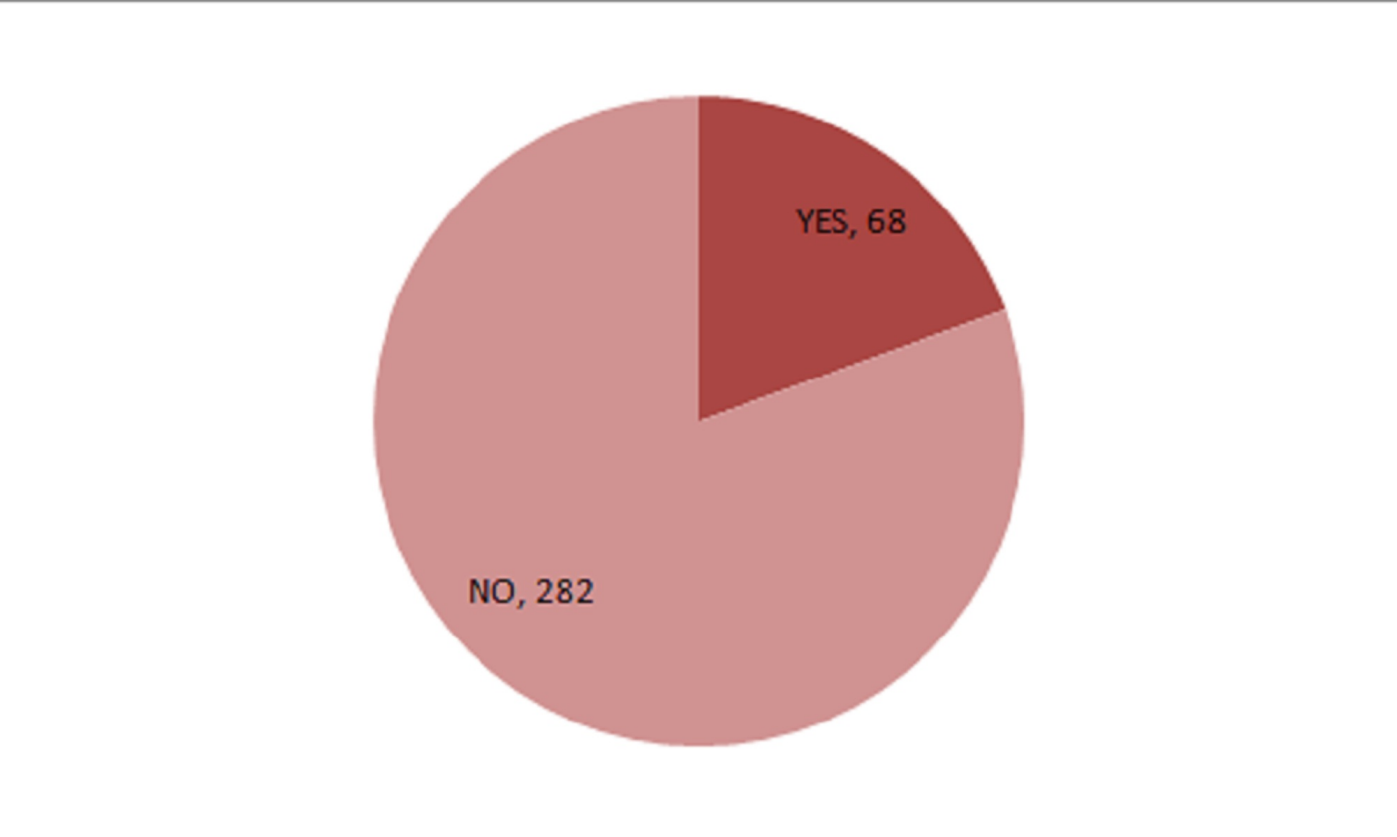
Neurological symptoms in COVID-19 patients.

**Figure 2:**
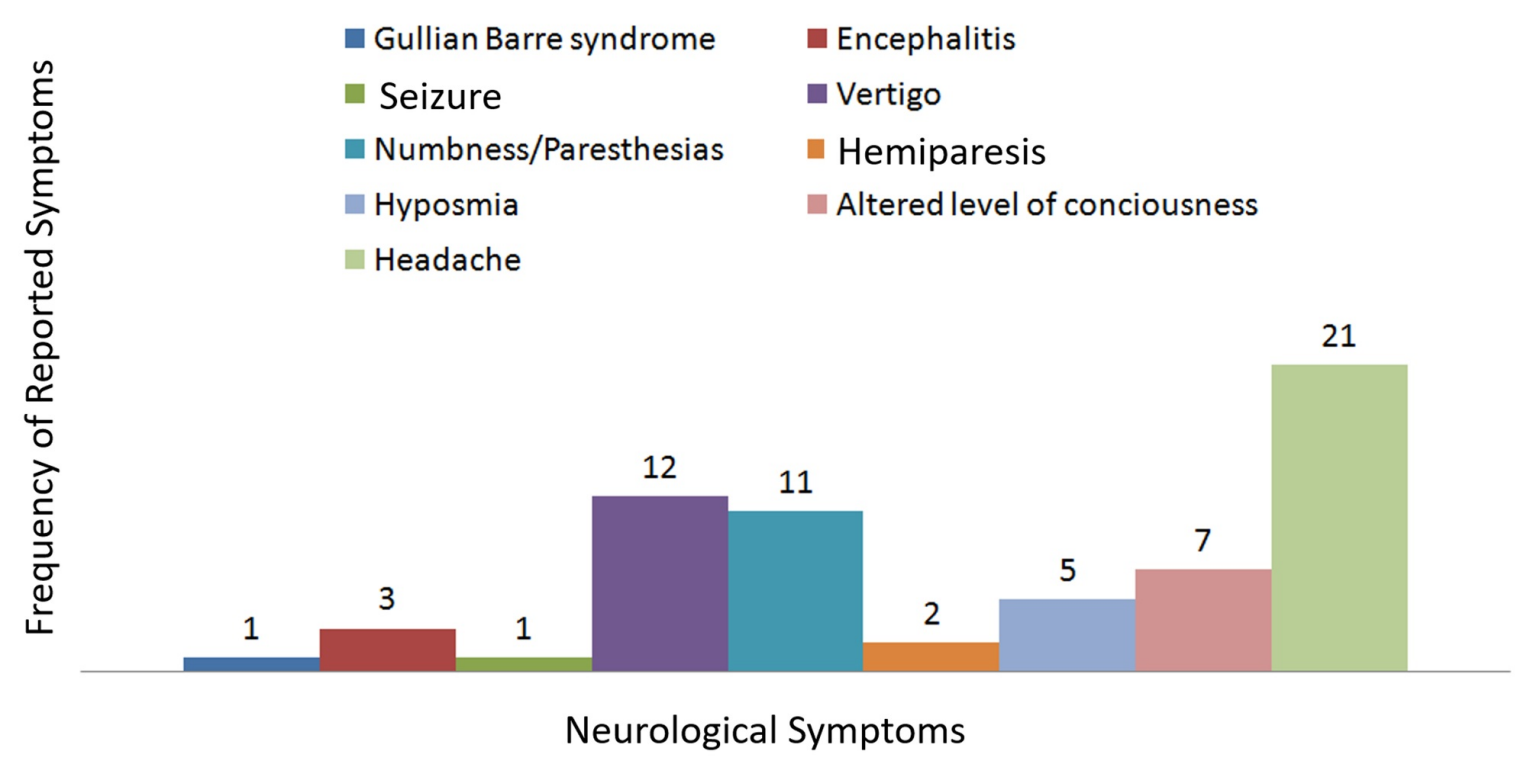
Frequency of individual neurological symptoms in COVID-19 patients.

The 350 patients included 245 (70%) men and 105 (30%) women; of these, 262 (74.9%) were married, and 88 (25.1%) were unmarried. Patients ranged in age from 17 to 88 years, with a mean ± SD age of 49.5 ± 17.4 years. Overall, 68 patients (18.9%) developed neurological manifestations (Figure 1). Headache was the most common neurological symptom, observed in 21 (6%) patients, followed by vertigo in 12 (3.4%), numbness/paresthesia in 11 (3.1%), altered level of consciousness in seven (2%), hyposmia/anosmia in five (1.4%), and encephalitis in three (0.9%) (Figure 2). Other symptoms included sudden hemiparesis (stroke) in two (0.6%) patients, flaccid paralysis due to GBS in one (0.3%), and seizure in one (0.3%). No significant association was found in neurological manifestation between COVID-19 patients aged <50 years and >50 years (Table 1).

**Table 1:** Neurological manifestation in COVID-19 patients aged <50 years and >50 years

Clinical Symptoms		Patient ages		P-value
		<50years	>50years	
Headache	Yes	12	9	0.124
	No	132	197	
Altered level of consciousness (ALOC)	Yes	1	6	0.145
	No	143	200	
Hyposmia/Anosmia	Yes	0	5	0.60
	No	144	201	
Sudden hemiparesis	Yes	0	2	0.236
	No	144	204	
Numbness/Paresthesia	Yes	6	5	0.359
	No	138	201	
Vertigo	Yes	7	5	0.218
	No	137	201	
Seizure	Yes	0	1	0.402
	No	144	205	
Encephalitis/Meningitis	Yes	0	3	0.402
	No	144	203	
Guillain-Barré syndrome (GBS)	Yes	0	1	0.402
	No	144	205	

## Discussion

SARS-CoV-2 is the cause of the COVID-19 pandemic. Although this virus primarily affects the respiratory system, it can also cause neurological manifestations, which may be predominant or even the presenting finding in some patients. This case series reviewed the clinical and laboratory data and outcome in 350 patients, consisting of both inpatients and self-isolated outpatients, who were PCR-positive for SARS-CoV-2 and were treated at Dow International Medical College, Dow University of Health Sciences, Karachi, Pakistan, from March to June 2020. Of these 350 patients, 68 (18.9%) presented with neurological manifestations. In comparison, of 214 COVID-19 patients in Wuhan, China, 78 (36.4%) had neurologic manifestation, a much higher percentage [2-5]. Our study confirmed that headache (6%), altered level of consciousness and encephalopathy (2%), hemiparesis (stroke; 0.6%), GBS (0.3%) and seizure (0.3%) were the most frequently reported neurological presentations [5,6,7,8].

Encephalitis

COVID-19 patients may present with neurological symptoms, including encephalitis and altered level of consciousness (encephalopathy). Genome sequencing showed that SARS-CoV-2 was present in the cerebrospinal fluid (CSF) of a Japanese patient who presented clinically with symptoms of meningoencephalitis [5]. In another case study, a woman presented with a three-day history of fever, cough, and altered mental status and was diagnosed with COVID-19 by detection of SARS-CoV-2 RNA in a nasopharyngeal swab [9]. In contrast, her CSF was negative for bacteria and viruses, and a computed tomography scan of the brain without contrast revealed symmetric bilateral hypointense signals in thalamus bilaterally, suggesting that this virus can cause encephalitis [9]. This may be due to increased expression of cytokines, including interleukin (IL)-2, IL-6, IL-7, tumor necrosis factor, granulocyte colony-stimulating factor, interferon-gamma, and free radicals associated with the severity of COVID-19.

Anosmia/hyposmia

Anosmia/hyposmia is one of the more common neurological manifestations of COVID-19 and may be the only presenting symptom in some patients [10]. A case study reported that a patient positive for SARS-CoV-2 presented with isolated sudden onset anosmia but no other symptoms of COVID-19 [11]. Anosmia/hyposmia presents mostly in patients in their early 20s and asymptomatic, healthy patients [12].

Cerebrovascular disease

The pathophysiology of hemiparesis (stroke) during for SARS-CoV-2 infection is multifactorial. Increased levels of acute-phase reactants, including leukocyte count and serum concentrations of C-reactive protein, D-dimer, ferritin, and lactate dehydrogenase, are the most common predictors of stroke. Viral infection of vascular endothelial cells accompanied by damage to the vasculature can predispose to infarct. Septicemia can predispose to coagulopathy, one of the manifestations of SARS-CoV-2 infection associated with hypercoagulability, and reduce the level of angiotensin-converting enzyme 2, resulting in tissue destruction leading to stroke [6,13]. The increased level of cytokines associated with SARS-CoV-2 infection is also the most potent cause of neuronal damage and stroke [14,15]. SARS-CoV-2 induced hypercoagulability may be the most important mechanism by which patients without any vascular risk factors develop cerebrovascular disease [16,17]. Timely diagnosis and management are vital in preventing morbidity and mortality in patients with acute stroke.

Guillain-Barré Syndrome

GBS is an immune-mediated demyelinating disorder to the peripheral nerves usually occurring after gastrointestinal or respiratory tract infection. Campylobacter jejuni, Zika virus, and influenza virus are the most frequent causes of GBS. Various neuromuscular disorders have been associated with COVID-19, as have other neurological disorders overlapping with GBS, such as Bickerstaff's encephalitis [7].

Seizure

Seizures (generalized tonic-clonic seizures) and altered levels of consciousness have been reported in COVID-10 patients. One study of 304 patients diagnosed with COVID-19 found that only two (0.7%) had developed seizures [8]. Risk factors for mortality in COVID-19 patients who require admission to hospital have been described [18].

The mechanism underlying the development of coronavirus-associated neurological complications remains unclear. These neurological manifestations may be due to the release of pro-inflammatory cytokines that predispose to vascular endothelial injury and increase the permeability of the blood-brain barrier [5]. A recent analysis of eight studies from China that included 46,248 infected patients found that hypertension (17%), diabetes mellitus (8%), and cardiovascular diseases (5%) were the most prevalent comorbidities [18]. Moreover, coronavirus-induced hypercoagulability may be the most potent mechanism for inducing cerebrovascular disease (stroke) in patients without any vascular risk factors [16,17].

The present study had several limitations, including its retrospective design and collection of data from a single center. Laboratory and radiological investigations were not performed in all patients, as many were self-isolated. Moreover, this study did not include long-term evaluations.

## Conclusions

Neurological manifestations of SARS-CoV-2-infected patients have not been documented during this pandemic. The present study evaluated the neurological manifestations of COVID-19 in patients in Karachi, Pakistan. The incidence of COVID-19 has grown dramatically around the world in recentmonths, and most cases are asymptomatic or mild and self-managed. Therefore, the actual numbers of cases are under-reported. The association of neurological manifestations with COVID-19 is still uncertain because many cases are also misdiagnosed as other febrile illnesses. Therefore, neurological manifestations of COVID-19 should be included in the differential diagnosis of patients with these neurological signs and symptoms. Diagnostic tests for SARS-CoV-2 should be performed in all patients with symptoms of respiratory illness and neurological symptoms.

Basic definitions and standard guidelines for research identifying the neurological manifestations of COVID-19 are warranted. Territorial, national, and global joint efforts by clinicians and researchers concentrated on high-caliber, straightforward, moral, and evidence-based exploratory practices would help push the worldwide health care network toward progress against this pandemic.

## Appendices

DEPARTMENT OF Medicine

DOW university of Health Science, DIMC Karachi

COVID-19 REGISTRATION FORM

Hospital MR # ___________________ Dated: _______________Time _______________ Name _____________ s/o d/o w/o ___________  Age & Sex:_________________ NIC # __________________  Profession_________________Home Address ______________                Contact #_______________________  Travel History/Contact History _______________________

                                                             Essential Criterion      

                                        Fever or measured temperature 100.4F of less than 14 days

        With any 1 of the following

Fever

Shortness of Breath,

Flu like illness

Severe Acute Respiratory Infection (SARI), pneumonia, Acute Respiratory Distress Infection, 

Sepsis and Septic Shock in a person requiring hospital admission, with no other etiology that fully explains the clinical presentation.

Neurologic clinical manifestations

·         Headache                          

·         Vertigo 

·         Hyposmia/Anosmia

·         Altered level of consciousness

·         Hemiparesis/Paraparesis

·         Parasthesia/Numbness

·         Encephalitis/meningitis

COVID-19 REGISTRATION FORM

___________________________                                                                 _____________________________

  Name of Data Entry Operator                                                                  Name/Stamp Signature of Doctor
